# Long noncoding RNA HOXA-AS2 promotes gastric cancer proliferation by epigenetically silencing P21/PLK3/DDIT3 expression

**DOI:** 10.18632/oncotarget.5599

**Published:** 2015-09-10

**Authors:** Min Xie, Ming Sun, Ya-nan Zhu, Rui Xia, Yan-wen Liu, Jie Ding, Hong-wei Ma, Xue-zhi He, Zhi-hong Zhang, Zhi-jun Liu, Xiang-hua Liu, Wei De

**Affiliations:** ^1^ Department of Biochemistry and Molecular Biology, Nanjing Medical University, Nanjing, People's Republic of China; ^2^ Department of Oncology, Second Affiliated Hospital, Nanjing Medical University, Nanjing, People's Republic of China; ^3^ Department of Pathology, First Affiliated Hospital, Nanjing Medical University, Nanjing, People's Republic of China

**Keywords:** gastric cancer, HOXA-AS2, proliferation, PRC2, P21/PLK3/DDIT3

## Abstract

Current evidence suggests that long noncoding RNAs (lncRNAs) may be an important class of functional regulators involved in human cancers development, including gastric cancer (GC). Here, we reported that HOXA cluster antisense RNA2 (HOXA-AS2), a 1048bp RNA, was upregulated in GC. Increased HOXA-AS2 expression in GC was associated with larger tumor size and higher clinical stage; patients with higher levels of HOXA-AS2 expression had a relatively poor prognosis. Further experiments revealed that HOXA-AS2 knockdown significantly inhibited GC cells proliferation by causing G1 arrest and promoting apoptosis, whereas HOXA-AS2 overexpression promoted cell growth. Furthermore, HOXA-AS2 could epigenetically repress the expression of P21, PLK3, and DDIT3 via binding with EZH2 (enhaner of zeste homolog 2), a key component of PRC2; ChIP assays demonstrated that EZH2 could directly bind to the promoter of P21, PLK3 and DDIT3, inducing H3K27 trimethylated. In conclusion, these data suggest that HOXA-AS2 could be an oncogene for GC partly through suppressing P21, PLK3, and DDIT3 expression; HOXA-AS2 may be served as a candidate prognostic biomarker and target for new therapies in human GC.

## INTRODUCTION

Gastric cancer (GC) is the fourth most commonly diagnosed cancer and the second leading cause of cancer death [1]. Although most patients diagnosed in early stage can be cured by chemotherapy, radiotherapy and surgical techniques, the survival rate of those diagnosed with advanced GC remains disappointed [2, 3]. Therefore, better understanding of the mechanism of gastric carcinogenesis is essential for GC diagnosis and therapy. In recent years, lots of oncogenes and tumor suppressors have been identified as key regulators for GC tumorigenesis and development, but the appropriate molecular biomarkers are rarely found. Thus, the exploration of novel useful indicators for GC diagnosis and treatment turns to be more important.

With the advance of high-resolution microarray and massively parallel sequencing technology, it has been gradually verified that only 2% of the genome sequences encode proteins, while the remainder is transcribed into noncoding RNAs (ncRNAs) [4, 5]. According to their size, ncRNAs can be extensively divided into two groups: short ncRNAs (<200nt) and long ncRNAs (>200nt) [6]. Among them, lncRNAs have limited or no protein-coding capacity, which are well known to regulate gene expression at multiple levels, including transcription and post-transcription regulation [7]. Importantly, aberrant expressions of lncRNAs may potentially alter basic cellular biological processes and contribute to tumorigenesis [8]. A plenty of evidence have demonstrated that lncRNAs act as crucial regulators in GC development and progression in recent years. For instance, lncRNA GAPLINC could regulate CD44-dependent cell invasiveness and associate with poor prognosis of GC [9]; lncRNA GHET1 promoted GC proliferation by increasing c-myc mRNA stability [10]. Our previous studies also showed that lncRNA ANRIL could be a growth regulator, which promoted GC proliferation by epigenetically silencing of miR-99a/miR-449a [11]; lncRNA HOTAIR could function as a competing endogenous RNA to regulate HER2 expression by sponging miR-331-3p in GC [12]. It is common that some molecular mechanisms of lncRNAs in GC have emerged from water, but the overall pathophysiological contributions of lncRNAs to GC remain unknown by now.

Given the importance of lncRNAs in GC, in the current study, we investigated lncRNA HOXA-AS2 (HOXA cluster antisense RNA2), which was previously shown to be an apoptosis repressor in all trans retinoic acid treated NB4 promyelocytic leukemia cells [13]. However, the biological functions and significance of HOXA-AS2 in other tumors including GC had not yet been established. In this study, we found that HOXA-AS2 expression was upregulated in GC, while showed significant downregulation or no significance in other commom types of tumors. We further discovered that HOXA-AS2 up-regulation was also correlated with larger tumor size, advanced pathologic stage and shorter overall survival of patients with GC. Moreover, functional analysis indicated that HOXA-AS2 could promote GC cell growth both *in vitro* and *in vivo* by epigenetically silencing P21, PLK3, and DDIT3 transcription via binding with EZH2. These results suggest that lncRNA HOXA-AS2 act as a non-coding oncogene in GC tumorigenesis and may be a potential biomarker for GC diagnosis and gene therapy.

## RESULTS

### HOXA-AS2 expression is upregulated in human GC tissues

**Figure 1:**
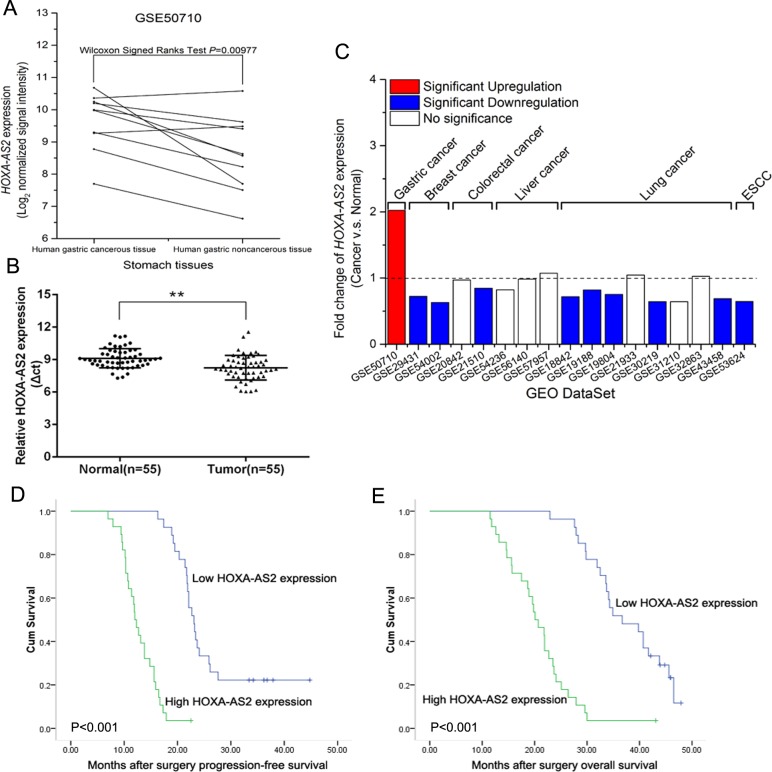
Relative HOXA-AS2 expression in GC tissues and its clinical significance **A.** Relative HOXA-AS2 expression in human gastric cancerous tissues (*n* = 10) compared with noncancerous tissue (*n* = 10) via GSE50710 data analysis (Wilcoxon signed Ranks Test, *P* = 0.00977). **B.** Relative expression of HOXA-AS2 in human GC tissues (*n* = 55) compared with corresponding non-tumor tissues (*n* = 55). HOXA-AS2 expression was examined by qRT-PCR and normalized to GAPDH expression (shown as Δct). **C.** Relative HOXA-AS2 expression in other types of cancer tissues compared with GC tissues by GEO DataSet analysis. HOXA-AS2 expressions were presented as the fold-change in tumor tissues relative to normal tissues. **D.**, **E.** Kaplan-Meier disease-free survival and overall survival curves according to HOXA-AS2 expression levels. Error bars indicate mean ± standard errors of the mean. *P < 0.05, **P < 0.01.

Firstly, we analyzed the expression levels of HOXA-AS2 in human GC tissues by using raw microarray data downloaded from GEO (GSE50710) [9], and found that HOXA-AS2 expression levels were upregulated in gastric cancerous tissues compared with noncancerous tissues (Figure 1A). Furthermore, qRT-PCR analysis was used to examine the expression of HOXA-AS2 in 55 paired GC samples and adjacent histologically normal tissues. Figure 1B showed that the HOXA-AS2 expressions were indeed higher in tumor tissues than in matched normal tissues. Next, we analyzed the expression levels of HOXA-AS2 in other types of cancers from GEO. Interestingly, HOXA-AS2 was significantly downregulated or represented no significance in breast cancer, colorectal cancer, liver cancer, lung cancer and esophagus cancer tissues (Figure 1C). These results imply that HOXA-AS2 overexpression in GC tissues contrary to other types of cancers may provide imperative clinical significance in GC diagnosis and therapy.

### Overexpression of HOXA-AS2 is associated with tumor size, TNM stage and poor prognosis of GC

To further understand the significance of HOXA-AS2 overexpression in GC, we examined the correlation between HOXA-AS2 expression and clinical pathological features. The clinical pathology findings of 55 gastric carcinoma patients were shown in Table 1. Noticeably, high HOXA-AS2 expression in GC was significantly correlated with tumor size (*p* = 0.007), advanced TNM stage (*p* = 0.011) and lymph node metastasis (*P* = 0.032). However, HOXA-AS2 expression was not associated with other parameters such as age (*p* = 0.423) and gender (*p* = 0.573) in GC (Table 1).

**Table 1:** Correlation between HOXA-AS2 expression and clinicopathological characteristics of gastric cancer patients

Characteristics	N(%)	HOXA-AS2		p Chi-squared test P-value
		High	Low	
**Gender**				0.573
Male	37(67.3%)	20	17	
Female	18(32.7%)	8	10	
**Age**				0.423
≤65	28(50.9%)	16	12	
> 65	27(49.1%)	12	15	
**Histological subtype**				0.285
Squamous cell carcinoma	28(50.9%)	12	16	
Adenocarcinoma	27(49.1%)	16	11	
**Stage**				0.011*
III	25(45.5%)	8	17	
III	30(54.5%)	20	10	
**Lymph node metastasis**				0.032*
Negative	17(30.9%)	5	12	
Positive	38(69.1%)	23	15	
**Tumor size**				0.007*
55 cm	15(27.3%)	3	12	
> 5 cm	40(72.7%)	25	15	

* P < 0.05 was considered significant (Chi-square test between 2 groups).

To evaluate the relationship between HOXA-AS2 expression level and outcome of GC patients after gastrectomy, disease-free survival (DFS) and overall survival (OS) curves were plotted according to HOXA-AS2 expression level by the Kaplan-Meier analysis and log-rank test, respectively (Figure 1D and 1E). According to the median ratio of relative HOXA-AS2 expression (1.9) in tumor tissues, the 55 gastric cancer patients were classified into two groups: relative high HOXA-AS2 group (*n* = 28, HOXA-AS2 expression ratio≥median ratio) and relative low HOXA-AS2 group (*n* = 27, HOXA-AS2 expression ratio≤median ratio). Remarkably, patients with high HOXA-AS2 expression level had poorer disease-free survival (*P* < 0.001) and overall survival (*P* < 0.001) (Figure 1D and 1E). These results imply that HOXA-AS2 overexpression may be useful in the development of novel prognostic or progression markers for GC.

### HOXA-AS2 promotes GC cell proliferation *in vitro*

To investigate the functional role of HOXA-AS2 in GC cells, we first performed qRT-PCR analysis to detect the expression of HOXA-AS2 in diverse human GC cell lines. As shown in Figure S1A, BGC-823, SGC-7901 and AGS cells expressed higher levels of HOXA-AS2 than other GC cells. We designed two different HOXA-AS2 siRNAs to transfect these three cell lines. QRT-PCR analysis was performed at 48h post-transfection and revealed that si-HOXA-AS2 1# had higher efficiency of interference than si-HOXA-AS2 2# (Figure S1B), So we chose si-HOXA-AS2 1# subsequently for the following experiments. Meanwhile, we induced ectopic overexpression of HOXA-AS2 by transfecting GC cell lines with pcDNA 3.1-HOXA-AS2 expression vector (Figure S1C).

**Figure 2:**
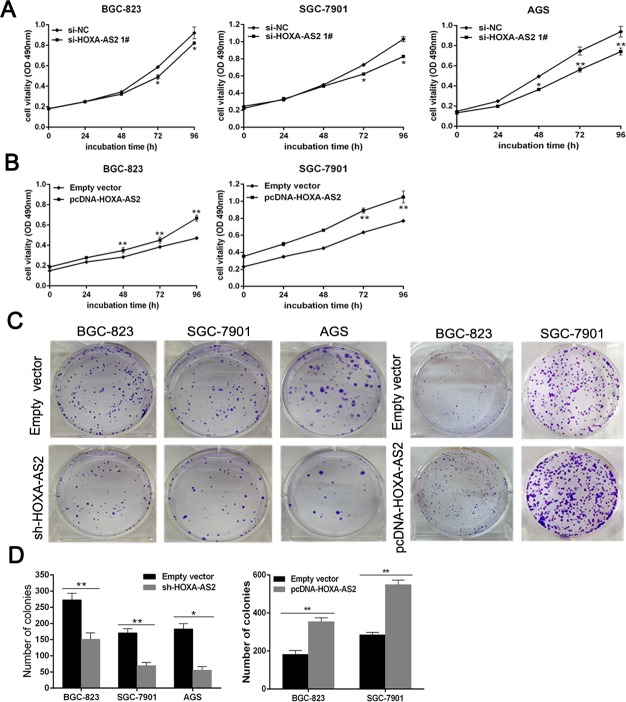
HOXA-AS2 promotes GC cell proliferation *in vitro* **A.** MTT assays were used to determine the viability of si-HOXA-AS2-transfected BGC-823, SGC-7901 and AGS cells. Experiments were performed in triplicate. **B.** MTT assays were performed to determine the viability of pcDNA-HOXA-AS2-transfected BGC-823 and SGC-7901 cells. Experiments were performed in triplicate. **C.** Colony formation assays were performed to determine the proliferation of sh-HOXA-AS2-transfected BGC-823, SGC-7901 and AGS cells or overexpression plasmid-transfected BGC-823 and SGC-7901 cells. Experiments were performed in triplicate. **D.** Colonies were counted and captured. Error bars indicate mean ± standard errors of the mean. *P < 0.05, **P < 0.01.

MTT assays showed that knockdown of HOXA-AS2 expression significantly inhibited cell growth in BGC-823, SGC-7901 and AGS cells compared with the respective controls (Figure 2A). Whereas, stimulated HOXA-AS2 expression promoted cell proliferation in BGC-823 and SGC-7901 cells (Figure 2B). Similarly, the results of colony-formation assays revealed that clonogenic survival was significantly decreased following downregulation of HOXA-AS2 in BGC-823, SGC-7901 and AGS cells, but markedly increased in HOXA-AS2 overexpression BGC-823 and SGC-7901 cells (Figure 2C and 2D). These data suggest that HOXA-AS2 may act as an oncogene involved in the promotion of GC cell proliferation.

### Downregulation of HOXA-AS2 promotes G1 arrest and causes apoptosis in GC cells

**Figure 3:**
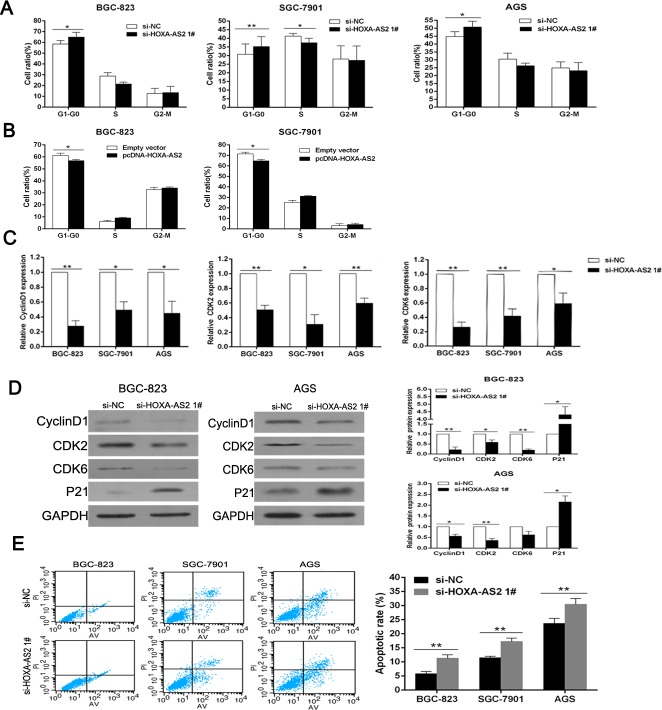
Downregulation of HOXA-AS2 promotes G1 arrest and causes apoptosis in GC cells **A.**, **B.** The bar chart represented the percentage of BGC-823, SGC-7901 and AGS cells in G0/G1, S or G2/M phase, as indicated. **C.** QRT-PCR analysis of CyclinD1, CDK2 and CDK6 after si-HOXA-AS2 or si-NC transfection in GC cells. **D.** Western blot analysis of CyclinD1, CDK2, CDK6 and P21 after si-HOXA-AS2 or si-NC transfection in BGC-823 and AGS cells. GAPDH protein was used as an internal control. **E.** Flow cytometry was used to detect the apoptotic rates of cells. LR, early apoptotic cells; UR, terminal apoptotic cells. Error bars indicate mean ± standard errors of the mean. *P < 0.05, **P < 0.01.

To further determine whether cell-cycle regulation was a potential contributing factor to cell growth by HOXA-AS2, cell-cycle progression was tested by flow cytometric analysis. The results showed that BGC-823, SGC-7901 and AGS cells transfected with si-HOXA-AS2 all had cell-cycle arrest at the G1-G0 phase compared with cells transfected with si-NC (Figure 3A). Inversely, overexpression of HOXA-AS2 led to a decrease of G1-G0 phase process and an increase of S phase process (Figure 3B). Moreover, qRT-PCR/western blot analysis showed that the mRNA and protein levels of CyclinD1/CDK2/CDK6 were significantly decreased in HOXA-AS2 si-RNA transfected cells (Figure 3C and 3D), confirming that HOXA-AS2 is involved in cell-cycle regulation.

In addition, we performed flow cytometry to examine whether GC cell proliferation was induced by cell apoptosis. The results revealed that the proportion of apoptotic cells following HOXA-AS2 siRNA treatment was increased (Figure 3E). Complementally, GSEA (GSE15459 [15], GSE51105 [16]) supported above phenomenon, which indicated that HOXA-AS2 was negatively correlated with intrinsic pathway for apoptosis, mitochondria pathway, caspase pathway and fas pathway (Figure S1D). These data suggested that HOXA-AS2-mediated promotion of GC cell proliferation seems to be mediated by regulation of the G1-S checkpoint and apoptosis.

### HOXA-AS2 epigenetically silences P21/PLK3/DDIT3 transcription by binding with EZH2

**Figure 4:**
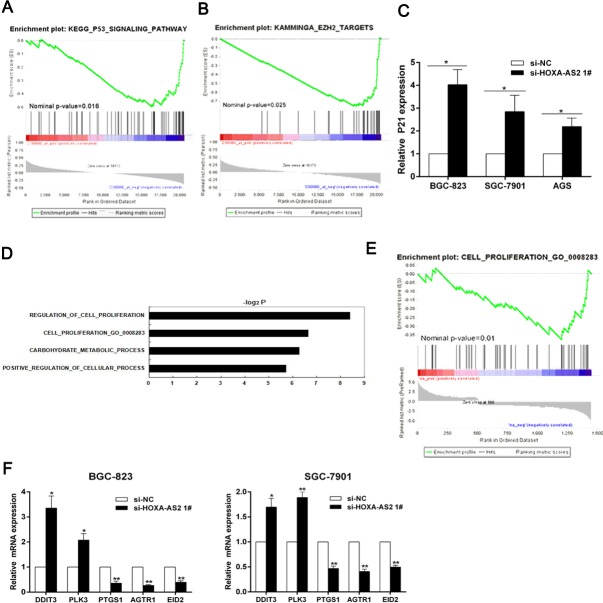
HOXA-AS2 knockdown increases the expression of genes involved in cell proliferation **A.**, **B.** HOXA-AS2 had significantly negative correlation with genes involved in P53 signaling pathway and the genes targeted by EZH2 in GC dataset (GSE15459). **C.** QRT-PCR was used to detect P21 mRNA levels after HOXA-AS2 knockdown in BGC-823, SGC-7901 and AGS cells. **D.** Gene Ontology analysis for all genes with altered expressions between si-NC and si-HOXA-AS2 treated BGC-823 cells *in vitro*. **E.** GSEA analysis indicated that cell proliferation process gene set were significantly enriched in genes upregulated by HOXA-AS2 knockdown. **F.** QRT-PCR was used to validate the changes of several mRNAs involved in cell proliferation. Error bars indicate mean ± standard errors of the mean. *P < 0.05, **P < 0.01.

To further explore the molecular mechanisms by which HOXA-AS2 contributes to the proliferation phenotype of GC cells, we conducted GSEA to identify potential downstream target genes. We found that HOXA-AS2 had significantly negative correlation with genes involved in P53 signaling pathway and the genes targeted by EZH2 [17] in one GC dataset (GSE15459) (Figure 4A and 4B). EZH2, a catalytic subunit of PRC2, catalyzed the trimethylation of histone H3 at lysine 27 (H3K27me3) [18, 19] and established a chromatin structure that was repressive to transcription [20]. According to these results, we focused on the cyclin-dependent kinase (CDK) inhibitor P21 for which was one of the most important downstream target genes of tumor suppressor P53. Moreover, Previous studies had demonstrated that P21 was an EZH2 target gene in cancer cells [21–23]. Subsequently, qRT-PCR and western blot analysis showed that HOXA-AS2 could inhibit P21 expressions both at mRNA and protein levels in GC cells (Figures 4C and 3D).

The suppression of p21 could partially explain the HOXA-AS2 function in cell cycle progression, but knockdown of HOXA-AS2 also induced apoptosis. To further explore its downstream targets partially responsible for HOXA-AS2 function in apoptosis, we performed microarray analysis in HOXA-AS2 knockdown BGC-823 cells and control cells. The differential expression analysis identified that 1451 genes were significantly differentially expressed (at least a two-fold change in expression) in cells after HOXA-AS2 knockdown. Of the differentially expressed genes, 509 were upregulated by at least 2-fold, and 942 were downregulated by at least 2-fold (Table S2). To further study the involved pathways activated by HOXA-AS2, we analyzed these genes using data collected from Gene Ontology (GO) database. We found that cell proliferation was involved in the affected biological process in HOXA-AS2 knockdown cells (Figure 4D). Next, GSEA analysis indicated that cell proliferation process gene set were significantly enriched in upregulated genes by HOXA-AS2 knockdown (Figure 4E). Subsequently, qRT-PCR was used to validate the changes of several mRNAs involved in cell apoptosis and proliferation (Figure 4F). On the basis of our microarray data, apoptosis related tumor suppressors PLK3 and DDIT3 were upregulated after HOXA-AS2 inhibition, which elucidated that these two genes may also be key downstream mediators of HOXA-AS2.

**Figure 5:**
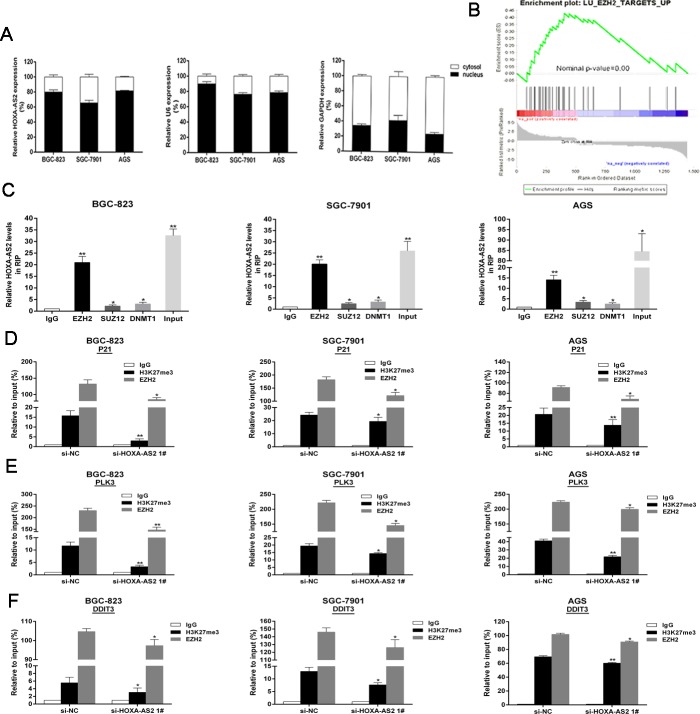
HOXA-AS2 epigenetically silences P21/PLK3/DDIT3 transcription by binding with EZH2 **A.** HOXA-AS2 expression levels in cell nucleus or cytoplasm of BGC-823, SGC-7901 and AGS cells were detected by qRT-PCR. U6 was used as a nucleus marker and GAPDH was used as a cytosol marker. **B.** Gene set containing EZH2-repressed genes were significantly enriched in genes upregulated by HOXA-AS2 knockdown. **C.** RIP experiments were performed in BGC-823, SGC-7901, AGS cells and the coprecipitated RNA was subjected to qRT-PCR for HOXA-AS2. The fold enrichment of HOXA-AS2 in EZH2/SUZ12/DNMT1 RIP is relative to its matching IgG control. **D.**, **E.** and **F.** ChIp-qRT-PCR of EZH2 occupancy and H3K27me3 binding in the P21/PLK3/DDIT3 promoters in BGC-823, SGC-7901, AGS cells treated with si-HOXA-AS2 (48h) or si-NC; IgG as a negative control. Error bars indicate mean ± standard errors of the mean. *P < 0.05, **P < 0.01.

In addition, we found HOXA-AS2 RNA were mostly located in the nucleus versus the cytosol (Figure 5A), thus suggesting HOXA-AS2 may exert transcriptional regulation function. GSEA analysis results showed that gene set containing EZH2-repressed genes were significantly enriched in upregulated genes by HOXA-AS2 knockdown [24] (Figure 5B). These results suggest that HOXA-AS2 may epigenetically inhibit downstream target genes by binding with EZH2. Next, we conducted RIP analysis to examine HOXA-AS2's binding with EZH2. As shown in Figure 5C, the endogenous HOXA-AS2 was enriched in the anti-EZH2 RIP fraction in BGC-823, SGC-7901 and AGS cells. HOTAIR, a known PRC2 associated lncRNA, was used as a positive control [25] (Figure S1E). However, we noticed that endogenous HOXA-AS2 was not yet combined with SUZ12 (Figure 5C). Given DNA-methylated regulation, we next performed anti-DNMT1 (DNA methyltransferase-1) RIP, and the result showed no combination between HOXA-AS2 and DNMT1 (Figure 5C). EZH2 siRNAs were transfected into BGC-823, SGC-7901 and AGS cells. The results showed that EZH2 expressions were effectively knocked down (Figure S1F), while P21, PLK3 and DDIT3 levels were up-regulated (Figure S1G).

Furthermore, the results of ChIP assays showed that EZH2 could directly bind to P21, PLK3 and DDIT3 promoter regions and induce H3K27me3 modification in BGC-823, SGC-7901 and AGS cells (Figure S2A, S2B and S2C). Knockdown of HOXA-AS2 resulted in reduced EZH2 binding and H3K27me3 occupany of P21, PLK3 and DDIT3 promoter locus (Figure 5D, 5E and 5F). These results suggest that HOXA-AS2 promotes GC cell growth partly through epigenetically silencing P21, PLK3 and DDIT3 transcription.

### Inhibition of P21 is potentially involved in the oncogene function of HOXA-AS2

**Figure 6:**
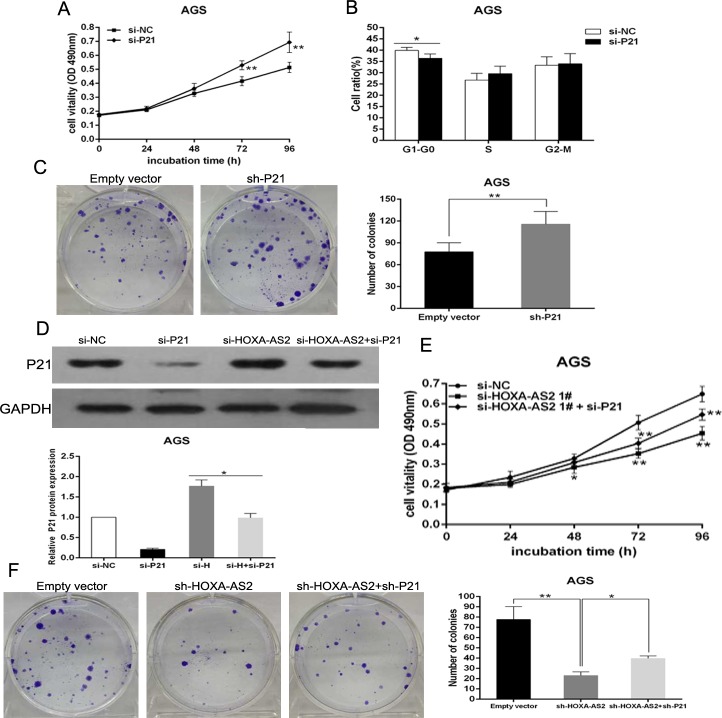
Down-regulation of p21 promotes AGS cells proliferation and is involved in the oncogene function of HOXA-AS2 AGS cells were transfected with si-P21 or co-transfected with si-HOXA-AS2 and si-P21. **A.** MTT assays were used to determine the cell viability for si-P21- transfected AGS cells. Experiments were performed in triplicate. **B.** Cell-cycle was detected by flow cytometry after AGS cells were transfected with si-P21. The bar chart represented the percentage of AGS cells in G0/G1, S or G2/M phase, as indicated. **C.** Colony-formation assays were used to determine the cell proliferation for sh-P21-transfected AGS cells. Experiments were performed in triplicate. **D.** The expression of P21 protein in AGS cells was analyzed by western blot. **E.**, **F.** MTT and Colony-formation assays were used to determine the cell viability for si-HOXA-AS2 and si-P21 co-transfected AGS cells. Experiments were performed in triplicate. Error bars indicate mean ± standard errors of the mean. *P < 0.05, **P < 0.01.

To validate the influence of P21 on cellular proliferation in GC cells, P21 expression was knocked down in AGS cells (Figure S1H). MTT and colony formation assays were then conducted to detect the cell viability. The results revealed that knockdown P21 expression could promote cellular proliferation (Figure 6A and 6C). Next, flow cytometry analysis indicated that downregulated expression of P21 decreased G1-G0 phase arrest (Figure 6B). These data showed that P21 inhibition could promote cell-cycle progression and proliferation of AGS cells, which was contrary to results of downregulated HOXA-AS2 in AGS cells.

To investigate whether P21 was involved in the HOXA-AS2-induced GC cell proliferation, we carried out rescue experiments. AGS cells were co-transfected with HOXA-AS2 and P21 siRNAs. We found that co-transfection could reduce the upregulated expression of P21 induced by the depletion of HOXA-AS2 (Figure 6D). MTT and colony formation assay results indicated that co-transfection could partially rescue si-HOXA-AS2 impaired proliferation in AGS cells (Figure 6E and 6F). These data indicate that HOXA-AS2 promotes GC cell proliferation partly through downregulation of P21 expression.

### HOXA-AS2 promotes tumorigenesis of GC cells *in vivo*

**Figure 7:**
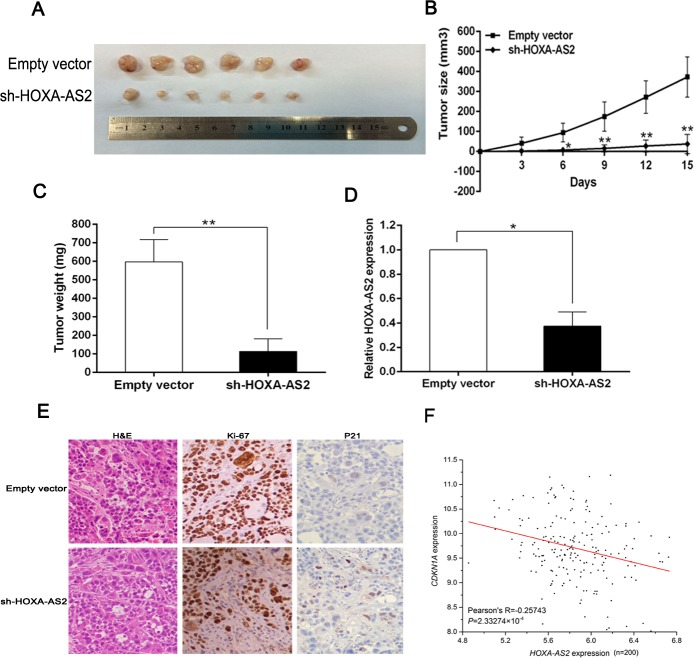
HOXA-AS2 promotes tumorigenesis of GC cells *in vivo* **A.** Empty vector or sh-HOXA-AS2 was transfected into SGC-7901 cells, which were injected in the nude mice (*n* = 6), respectively. Tumors formed in sh-HOXA-AS2 group were dramatically smaller relative to controls. **B.** Tumor volumes were calculated after injection every three days. Points, mean (*n* = 6); bars indicate SD. **C.** Tumor weights were represented as means of tumor weights ± SD. **D.** QRT-PCR was performed to detect the average expression of HOXA-AS2 in xenograft tumors (*n* = 6). **E.** The tumor sections were under H&E staining and IHC staining using antibodies against ki-67 and P21. **F.** correlation analysis revealed that HOXA-AS2 expression levels were inversely correlated with P21 expression levels in GC gene expression dataset GSE15459. Error bars indicate mean ± standard errors of the mean. *P < 0.05, **P < 0.01.

To determine whether the level of HOXA-AS2 expression could affect tumorigenesis, sh-HOXA-AS2 and empty vector transfected SGC-7901 cells were inoculated into nude mice. All mice developed xenograft tumors at the injection site. 15 days after injection, we found that the tumors formed in the sh-HOXA-AS2 group were generally smaller than those in the control group (Figure 7A). Moreover, tumor growth in sh-HOXA-AS2 group was significantly slower than that in the empty vector group (Figure 7B). Additionally, the average tumor weight was obviously lower in the sh-HOXA-AS2 group compared with the empty vector group (Figure 7C). QRT-PCR analysis found that the levels of HOXA-AS2 expression in tumor tissues formed from sh-HOXA-AS2 cells were lower than in tumors formed in the control group (Figure 7D). The tumors developed from sh-HOXA-AS2 cells displayed lower Ki-67 staining and higher P21 staining than that in tumors formed from empty vector transfected cells (Figure 7E). Finally, correlation analysis revealed that HOXA-AS2 expression levels were inversely correlated with P21 expression levels in GC gene expression dataset GSE15459 (Figure 7F, Table S3). These results indicate that HOXA-AS2 overexpression is significantly associated with the *in vivo* proliferation capacity of GC cells and suppressed P21 expression *in vivo.*

## DISCUSSION

Over the past decades, more and more studies have emphasized the emerging significance of lncRNAs in diverse human diseases including cancers [26]. Recently, more evidence have emerged that dysregulation of many lncRNAs in GC could be considered as one of the leading forces during GC tumorigenesis, as exemplified by GAS5 in our previous study [27]. Therefore, identification of GC-associated lncRNAs and investigation of their biological functions and clinical significance may provide a strategy and faciliate the development of lncRNA-directed diagnosis and prognosis against this malignancy. In this study, we ascertained that the expression of HOXA-AS2 was upregulated in GC tissues, while significantly downregulated or revealed no expressional difference in other common types of cancer tissues, suggesting that HOXA-AS2 may be an independent clinical marker in GC prognosis and therapy. Additionally, HOXA-AS2 knockdown could significantly inhibit GC cell proliferation both *in vitro* and *in vivo* by causing G1 arrest and promoting apoptosis.

The importance of lncRNAs in human cancer may be associated with their abilities to impact cellular functions through various mechanisms. lncRNAs can act as molecular decoys, which bind and titrate away proteins or RNAs to indirectly exert biological functions in multiple kingdoms of life. For instance, lncRNA MALAT1 could bind to SFPQ, thus releasing PTBP2 from the SFPQ/PTBP2 complex; the increased SFPQ-detached PTBP2 promoted CRC cell proliferation and migration [28]. In addition, lncRNAs can recruit chromatin-modifying enzymes to target genes by acting as guides, either in cis (near the site of lncRNA production) [29] or in trans to distant target genes [30]. Occasionally, some lncRNAs can serve as molecular scaffolds to bind relevant molecular components to regulate gene expression [31, 32]. In this study, we found that HOXA-AS2 could recruit and bind with EZH2 not SUZ12. It is evident that more than 20% of lncRNAs are bound by PRC2 in various cells, and silence downstream target genes [33, 34]. EZH2, a key catalytic subunit of PRC2, functions as a histone methyltransferase that specifically induces histone H3 lysine 27 trimethylation (H3K27me3) to target genes [35]; EZH2 overexpression plays an important role in cancer development and progression [36, 37].

GSEA analysis indicated that P21 was a possible target of HOXA-AS2; simultaneously, microarray data showed that PLK3 and DDIT3 also might be downstream mediators of HOXA-AS2. The cyclin-dependent kinase (CDK) inhibitor P21, one of the most crucial downstream target genes of P53 signaling pathway, is expressed ubiquitously and plays multiple roles in many cellular processes during unperturbed cell growth. The critical well-known function of P21 is to block cell proliferation in normal and cancer cells by inhibiting the activity of kinases important for G1/S transition such as CyclinD/CDK4, CyclinD/CDK6 and CyclinE/CDK2 [38–40]. Meanwhile, P21 is a critical molecule for inhibiting cell proliferation in normal and cancer cells [41]. Rescue experiments verified that inhibition of P21 is potentially involved in the oncogene function of HOXA-AS2. PLK3, a cytokine-inducible kinase, prevents cell-cycle progression and tumorigenesis. In most studies, deregulated expression of Plk3 results in cell-cycle arrest, apoptosis and growth suppression [42, 43]. DDIT3 has been reported as a pro-apoptotic factor in endoplasmic reticulum stress, DNA damage and repair biological process [44, 45]. DDIT3 was also viewed as a promising target in the anticancer research, for the identification of its tumor suppressor functions [46, 47]. In the present study, we found that P21, PLK3, and DDIT3 were epigenetically silenced by HOXA-AS2-EZH2 complex in GC cells.

In summary, we have shown that HOXA-AS2 was upregulated in GC tissues and its upregulation may be associated with GC patients poor prognosis for the first time. The promotion of HOXA-AS2 on GC cell proliferation and tumorigenesis may partly via epigenetically silencing P21, PLK3 and DDIT3 transcription by binding with EZH2. Collectively, our study may provide a new perspective that lncRNA HOXA-AS2 act as a non-coding oncogene in GC tumorigenesis and a novel target for early diagnosis and treatment of GC. However, the other possible mechanisms by which HOXA-AS2 participates in GC cell biological function remains to be fully understood.

## MATERIALS AND METHODS

### Differential expression analysis

GC gene expression data were downloaded from the Gene Expression Omnibus (GEO) dataset. The independent data sets from GSE50710, GSE29431, GSE54002, GSE20842, GSE21510, GSE54236, GSE56140, GSE57957, GSE18842, GSE19188, GSE19804, GSE21933, GSE30219, GSE31210, GSE32863, GSE43458, and GSE53624 were included in this study. The raw CEL files were downloaded from GEO database and normalized using Robust Multichip Average (RMA). After we downloaded probe sequences from GEO or microarray manufacturers, blast+2.2.30 was used to re-annotates probe on GENCODE Release 21 sequence databases for lncRNA and mRNA. For multiple probes corresponding to one gene, maximum normalized signal was selected to generate expressions of lncRNA and mRNA.

### Tissue collection

55 paired GC and corresponding adjacent non-tumorous gastric samples were obtained from patients who underwent surgery at First Affiliated Hospital of Nanjing Medical University between 2010 and 2011. All cases were confirmed as GC based on histopathological evaluation. The clinicopathological characteristics of the GC patients are summarized in Table 1. No local or systemic treatment was conducted in these patients before surgery. All collected tissue samples were immediately snap-frozen in liquid nitrogen and stored at −80°C until required. Our study was approved by the Research Ethics Committee of Nanjing Medical University, China. Written informed consent was obtained from all patients.

### Cell culture

Three human GC cell lines (BGC823, SGC7901, AGS) were purchased from the Institute of Biochemistry and Cell Biology of the Chinese Academy of Sciences (Shanghai, China). BGC-823 cells were cultured in RPMI 1640; SGC-7901 cells were cultured in DMEM (GIBCO-BRL) medium; AGS cells were cultured in F12 medium supplemented with 10% fetal bovine serum (FBS), 100 U/ml penicillin and 100 mg/ml streptomycin (Invitrogen, Carlsbad, CA, USA) in humidified air at 37°C with 5% CO2.

### RNA extraction and qRT-PCR assays

Total RNA was extracted from tissues or cultured cells using TRIZOL reagent (Invitrogen). For qRT-PCR, RNA was reverse transcribed to cDNA by using a Reverse Transcription Kit (Takara, Dalian, China). Real-time PCR analyses were performed with SYBR Premix Ex Taq (Takara, Dalian China). Results were normalized to the expression of GAPDH. The specific primers used were shown in Table S1. The qRT-PCR assays were conducted on an ABI 7500, and data collected with this instrument. Our qRT-PCR results were analyzed and expressed relative to threshold cycle (CT) values, and then converted to fold changes.

### Transfection of gastric cancer cells

GC cells were transfected with siRNAs and plasmid vectors using Lipofectamine 2000 (Invitrogen, USA), according to the manufacturer's protocol. Two individual HOXA-AS2 siRNAs (si-HOXA-AS2 1# and 2#), P21 siRNA, EZH2 siRNA and scrambled negative control siRNA (si-NC) were purchased from Invitrogen. Plasmid vectors (sh-HOXA-AS2, sh-P21 and empty vector) for transfection were extracted by DNA Midiprep kit (Qiagen, Hilden, Germany). The nucleotide sequences of siRNAs for HOXA-AS2, P21 and EZH2 were shown in Table S1. The full-length complementary DNA of HOXA-AS2 was synthesized by Realgene (Nanjing, China) and subcloned into the pcDNA3.1 (+) vector (Invitrogen) according to the manufacturer's instructions. At 48 h post-transfection, cells were harvested for qRT-PCR or western blot analysis.

### Cell proliferation assays

Cell viability was tested with a Cell Proliferation Reagent Kit I (MTT) (Roche Applied Science). The BGC-823, SGC-7901 and AGS cells transfected with si-HOXA-AS2 or pcDNA-HOXA-AS2 (3000 cells/well) were grown in 96-well plates. Cell viability was assessed every 24h following the manufacturer's protocol. All experiments were performed in quadruplicate. For colony formation assay, a certain number of transfected cells were placed in each well of 6-well plates and maintained in proper media containing 10% FBS for two weeks, during which the medium was replaced every 4 days. After 14 days, the cells were fixed with methanol and stained with 0.1% crystal violet (Sigma-Aldrich). Visible colonies were then counted. For each treatment group, wells were assessed in triplicate, and experiments were independently repeated three times.

### Flow-cytometric analysis

BGC-823, SGC-7901 and AGS cells transfected with si-HOXA-AS2 or pcDNA-HOXA-AS2 were harvested 48 hr after transfection by trypsinization. After the double staining with FITC-Annexin V and Propidium iodide (PI) was done using the FITC Annexin V Apoptosis Detection Kit (BD Biosciences) according to the manufacturer's recommendations, the cells were analyzed with a flow cytometry (FACScan^®^; BD Biosciences) equipped with a CellQuest software (BD Biosciences). Cells were discriminated into viable cells, dead cells, early apoptotic cells, apoptotic cells, and then the relative ratio of early apoptotic cells were compared to control transfectant from each experiment. Cells for cell cycle analysis were stained with PI using the CycleTEST™ PLUS DNA Reagent Kit (BD Biosciences) following the protocol and analyzed by FACScan. The percentage of the cells in G0/G1, S, and G2/M phase were counted and compared.

### Western blot assay and antibodies

Cells protein lysates were separated by 10% SDS-polyacrylamide gel electrophoresis (SDS-PAGE), transferred to 0.22μm NC membranes (Sigma) and incubated with specific antibodies. ECL chromogenic substrate was used to were quantified by densitometry (Quantity One software; Bio-Rad). GAPDH antibody was used as control, and Anti-P21, CyclinD1, CDK2, CDK6 (1:1000) were purchased from Cell Signaling Technology, Inc (CST).

### GSEA

Gene Set Enrichment analysis (GSEA) software was downloaded from Broad Institute (http://www.broadinstitute.org/gsea/index.jsp). Significantly enriched gene sets were identified, which produced a norminal P-value 0.05.

### Microarray data analysis

RNA extraction and purification, RNA amplification and labeling, array hybridization, and data acquisition were performed as described in the Affymetrix manufacturer's instructions. Affymetrix Human U133 Plus 2.0 whole genome chips were used (Santa Clara, CA, US). Array hybridization and wash was performed using GeneChip^®^ Hybridization, Wash and Stain Kit (Cat#900720, Affymetrix, Santa Clara, CA, US) in Hybridization Oven 645 (Cat#00-0331-220V, Affymetrix, Santa Clara, CA, US) and Fluidics Station 450 (Cat#00-0079, Affymetrix, Santa Clara, CA, US) followed the manufacturer's instructions. Slides were scanned by GeneChip^®^ Scanner 3000 (Cat#00-00212, Affymetrix, Santa Clara, CA, US) and Command Console Software 4.0 (Affymetrix, Santa Clara, CA, US) with default settings. Raw data were normalized by MAS 5.0 algorithm, Gene Spring Software 11.0 (Agilent technologies, Santa Clara, CA, US).

### Subcellular fractionation location

The separation of nuclear and cytosolic fractions was performed using the PARIS Kit (Life Technologies) according to the manufacturer's instructions.

### RNA immunoprecipitation (RIP) assay

RNA immunoprecipitation was performed using the EZMagna RIP kit (Millipore, Billerica, MA, USA) following the manufacturer's protocol. BGC-823, SGC-7901 and AGS cells at 80-90% confluency were scraped off, then lysed in complete RIP lysis buffer. 100 μl of whole cell extract was incubated with RIP buffer containing magnetic beads conjugated with antibodies that recognized EZH2, SUZ12, DNMT1 or with control IgG (millipore) for 6hr at 4°C. After the beads were washed with wash buffer, the complexes were incubated with 0.1% SDS/0.5 mg/ml Proteinase K (30 min at 55°C) to remove proteins, respectively. The RNA concentration was measured using a NanoDrop (Thermo Scientific) and the RNA quality assessed using a bioanalyser (Agilent, Santa Clara, CA, USA). Furthermore, purified RNA was subjected to qRT-PCR analysis to demonstrate the presence of HOXA-AS2 and HOTAIR using specific primers.

### Chromatin immunoprecipitation

BGC-823, SGC-7901 and AGS cells were treated with formaldehyde and incubated for 10 mins to generate DNA-protein cross-links. Cell lysates were then sonicated to generate chromatin fragments of 200-300 bp and immunoprecipitated with EZH2 and H3K27me3-specific antibody (CST) or IgG as control. Precipitated chromatin DNA was recovered and analyzed by qRT-PCR.

### Tumor formation assay in a nude mouse model

Female athymic BALB/c nude mice (4-weeks-old) were maintained under pathogen-free conditions and manipulated according to protocols approved by the Shanghai Medical Experimental Animal Care Commission. SGC-7901 cells were stably transfected with sh-HOXA-AS2, empty vector and harvested from 6-well cell culture plates, washed with phosphate-buffered saline (PBS), and re-suspended at a concentration of 1 × 108 cells/ml. A total of 100 μL of suspended cells was subcutaneously injected into a single side of the posterior flank of each mouse. Tumor growth was examined every 3 days, and tumor volumes were calculated using the equation V = 0.5 × D × d2 (V, volume; D, longitudinal diameter; d, latitudinal diameter). At 15 days post-injection, mice were euthanized, and the subcutaneous growth of each tumor was examined. This study was carried out in strict accordance with the recommendations in the Guide for the Care and Use of Laboratory Animals of the National Institutes of Health. The protocol was approved by the Committee on the Ethics of Animal Experiments of the Nanjing medical University.

### Immunohistochemical (IHC) analysis

The primary tumors were immunostained for Ki-67 and P21 as previously described [14].

### Statistical analysis

The Students t test (2 tailed), one-way ANOVA, and Mann-Whitney U test were conducted to analyze the in vitro and in vivo data by SPSS 16.0 software. P values less than 0.05 were considered significant.

## SUPPLEMENTARY MATERIAL FIGURES AND TABLES









## References

[1] Herszenyi L, Tulassay Z (2010). Epidemiology of gastrointestinal and liver tumors. EUR REV MED PHARMACOL SCI.

[2] Wang XN, Liang H (2010). Some problems in the surgical treatment of gastric cancer. CHIN J CANCER.

[3] Saka M, Morita S, Fukagawa T, Katai H (2011). Present and future status of gastric cancer surgery. JPN J CLIN ONCOL.

[4] Esteller M (2011). Non-coding RNAs in human disease. NAT REV GENET.

[5] Ponting CP, Oliver PL, Reik W (2009). Evolution and functions of long noncoding RNAs. CELL.

[6] An integrated encyclopedia of DNA elements in the human genome (2012). NATURE.

[7] Kung JT, Colognori D, Lee JT (2013). Long noncoding RNAs: past, present, and future. GENETICS.

[8] Gutschner T, Diederichs S (2012). The hallmarks of cancer: a long non-coding RNA point of view. RNA BIOL.

[9] Hu Y, Wang J, Qian J, Kong X, Tang J, Wang Y, Chen H, Hong J, Zou W, Chen Y, Xu J, Fang JY (2014). Long noncoding RNA GAPLINC regulates CD44-dependent cell invasiveness and associates with poor prognosis of gastric cancer. CANCER RES.

[10] Yang F, Xue X, Zheng L, Bi J, Zhou Y, Zhi K, Gu Y, Fang G (2014). Long non-coding RNA GHET1 promotes gastric carcinoma cell proliferation by increasing c-Myc mRNA stability. FEBS J.

[11] Zhang EB, Kong R, Yin DD, You LH, Sun M, Han L, Xu TP, Xia R, Yang JS, De W, Chen J (2014). Long noncoding RNA ANRIL indicates a poor prognosis of gastric cancer and promotes tumor growth by epigenetically silencing of miR-99a/miR-449a. ONCOTARGET.

[12] Liu XH, Sun M, Nie FQ, Ge YB, Zhang EB, Yin DD, Kong R, Xia R, Lu KH, Li JH, De W, Wang KM, Wang ZX (2014). Lnc RNA HOTAIR functions as a competing endogenous RNA to regulate HER2 expression by sponging miR-331-3p in gastric cancer. MOL CANCER.

[13] Zhao H, Zhang X, Frazao JB, Condino-Neto A, Newburger PE (2013). HOX antisense lincRNA HOXA-AS2 is an apoptosis repressor in all trans retinoic acid treated NB4 promyelocytic leukemia cells. J CELL BIOCHEM.

[14] Liao WT, Wang X, Xu LH, Kong QL, Yu CP, Li MZ, Shi L, Zeng MS, Song LB (2009). Centromere protein H is a novel prognostic marker for human nonsmall cell lung cancer progression and overall patient survival. CANCER-AM CANCER SOC.

[15] Wu Y, Grabsch H, Ivanova T, Tan IB, Murray J, Ooi CH, Wright AI, West NP, Hutchins GG, Wu J, Lee M, Lee J, Koo JH, Yeoh KG, van Grieken N, Ylstra B (2013). Comprehensive genomic meta-analysis identifies intra-tumoural stroma as a predictor of survival in patients with gastric cancer. GUT.

[16] Busuttil RA, George J, Tothill RW, Ioculano K, Kowalczyk A, Mitchell C, Lade S, Tan P, Haviv I, Boussioutas A (2014). A signature predicting poor prognosis in gastric and ovarian cancer represents a coordinated macrophage and stromal response. CLIN CANCER RES.

[17] Kamminga LM, Bystrykh LV, de Boer A, Houwer S, Douma J, Weersing E, Dontje B, de Haan G (2006). The Polycomb group gene Ezh2 prevents hematopoietic stem cell exhaustion. BLOOD.

[18] Margueron R, Reinberg D (2011). The Polycomb complex PRC2 and its mark in life. NATURE.

[19] Simon JA, Kingston RE (2013). Occupying chromatin: Polycomb mechanisms for getting to genomic targets, stopping transcriptional traffic, and staying put. MOL CELL.

[20] Yuan W, Wu T, Fu H, Dai C, Wu H, Liu N, Li X, Xu M, Zhang Z, Niu T, Han Z, Chai J, Zhou XJ, Gao S, Zhu B (2012). Dense chromatin activates Polycomb repressive complex 2 to regulate H3 lysine 27 methylation. SCIENCE.

[21] Seward S, Semaan A, Qazi AM, Gruzdyn OV, Chamala S, Bryant CC, Kumar S, Cameron D, Sethi S, Ali-Fehmi R, Morris R, Bouwman DL, Munkarah AR, Weaver DW, Gruber SA, Batchu RB (2013). EZH2 blockade by RNA interference inhibits growth of ovarian cancer by facilitating re-expression of p21(waf1/cip1) and by inhibiting mutant p53. CANCER LETT.

[22] Chen J, Li J, Han Q, Sun Z, Wang J, Wang S, Zhao RC (2012). Enhancer of zeste homolog 2 is overexpressed and contributes to epigenetic inactivation of p21 and phosphatase and tensin homolog in B-cell acute lymphoblastic leukemia. Exp Biol Med (Maywood).

[23] Bai J, Chen J, Ma M, Cai M, Xu F, Wang G, Tao K, Shuai X (2014). Inhibiting enhancer of zeste homolog 2 promotes cellular senescence in gastric cancer cells SGC-7901 by activation of p21 and p16. DNA CELL BIOL.

[24] Lu C, Han HD, Mangala LS, Ali-Fehmi R, Newton CS, Ozbun L, Armaiz-Pena GN, Hu W, Stone RL, Munkarah A, Ravoori MK, Shahzad MM, Lee JW, Mora E, Langley RR, Carroll AR (2010). Regulation of tumor angiogenesis by EZH2. CANCER CELL.

[25] Gupta RA, Shah N, Wang KC, Kim J, Horlings HM, Wong DJ, Tsai MC, Hung T, Argani P, Rinn JL, Wang Y, Brzoska P, Kong B, Li R, West RB, van de Vijver MJ (2010). Long non-coding RNA HOTAIR reprograms chromatin state to promote cancer metastasis. NATURE.

[26] Gibb EA, Brown CJ, Lam WL (2011). The functional role of long non-coding RNA in human carcinomas. MOL CANCER.

[27] Sun M, Jin FY, Xia R, Kong R, Li JH, Xu TP, Liu YW, Zhang EB, Liu XH, De W (2014). Decreased expression of long noncoding RNA GAS5 indicates a poor prognosis and promotes cell proliferation in gastric cancer. BMC CANCER.

[28] Ji Q, Zhang L, Liu X, Zhou L, Wang W, Han Z, Sui H, Tang Y, Wang Y, Liu N, Ren J, Hou F, Li Q (2014). Long non-coding RNA MALAT1 promotes tumour growth and metastasis in colorectal cancer through binding to SFPQ and releasing oncogene PTBP2 from SFPQ/PTBP2 complex. Br J Cancer.

[29] Wang KC, Yang YW, Liu B, Sanyal A, Corces-Zimmerman R, Chen Y, Lajoie BR, Protacio A, Flynn RA, Gupta RA, Wysocka J, Lei M, Dekker J, Helms JA, Chang HY (2011). A long noncoding RNA maintains active chromatin to coordinate homeotic gene expression. NATURE.

[30] Huarte M, Guttman M, Feldser D, Garber M, Koziol MJ, Kenzelmann-Broz D, Khalil AM, Zuk O, Amit I, Rabani M, Attardi LD, Regev A, Lander ES, Jacks T, Rinn JL (2010). A large intergenic noncoding RNA induced by p53 mediates global gene repression in the p53 response. CELL.

[31] Kotake Y, Nakagawa T, Kitagawa K, Suzuki S, Liu N, Kitagawa M, Xiong Y (2011). Long non-coding RNA ANRIL is required for the PRC2 recruitment to and silencing of p15(INK4B) tumor suppressor gene. ONCOGENE.

[32] Yap KL, Li S, Munoz-Cabello AM, Raguz S, Zeng L, Mujtaba S, Gil J, Walsh MJ, Zhou MM (2010). Molecular interplay of the noncoding RNA ANRIL and methylated histone H3 lysine 27 by polycomb CBX7 in transcriptional silencing of INK4a. MOL CELL.

[33] Zhao J, Ohsumi TK, Kung JT, Ogawa Y, Grau DJ, Sarma K, Song JJ, Kingston RE, Borowsky M, Lee JT (2010). Genome-wide identification of polycomb-associated RNAs by RIP-seq. MOL CELL.

[34] Khalil AM, Guttman M, Huarte M, Garber M, Raj A, Rivea MD, Thomas K, Presser A, Bernstein BE, van Oudenaarden A, Regev A, Lander ES, Rinn JL (2009). Many human large intergenic noncoding RNAs associate with chromatin-modifying complexes and affect gene expression. Proc Natl Acad Sci U S A.

[35] Cao R, Wang L, Wang H, Xia L, Erdjument-Bromage H, Tempst P, Jones RS, Zhang Y (2002). Role of histone H3 lysine 27 methylation in Polycomb-group silencing. SCIENCE.

[36] Varambally S, Cao Q, Mani RS, Shankar S, Wang X, Ateeq B, Laxman B, Cao X, Jing X, Ramnarayanan K, Brenner JC, Yu J, Kim JH, Han B, Tan P, Kumar-Sinha C (2008). Genomic loss of microRNA-101 leads to overexpression of histone methyltransferase EZH2 in cancer. SCIENCE.

[37] Ning X, Shi Z, Liu X, Zhang A, Han L, Jiang K, Kang C, Zhang Q (2015). DNMT1 and EZH2 mediated methylation silences the microRNA-200b/a/429 gene and promotes tumor progression. CANCER LETT.

[38] Gartel AL, Radhakrishnan SK (2005). Lost in transcription: p21 repression, mechanisms, and consequences. CANCER RES.

[39] Sherr CJ, Roberts JM (1995). Inhibitors of mammalian G1 cyclin-dependent kinases. GENES DEV.

[40] Sherr CJ, Roberts JM (1999). CDK inhibitors: positive and negative regulators of G1-phase progression. GENES DEV.

[41] Abbas T, Dutta A (2009). p21 in cancer: intricate networks and multiple activities. NAT REV CANCER.

[42] Conn CW, Hennigan RF, Dai W, Sanchez Y, Stambrook PJ (2000). Incomplete cytokinesis and induction of apoptosis by overexpression of the mammalian polo-like kinase, Plk3. CANCER RES.

[43] Iida M, Sasaki T, Komatani H (2009). Overexpression of Plk3 causes morphological change and cell growth suppression in Ras pathway-activated cells. J BIOCHEM.

[44] Yoon MJ, Kang YJ, Lee JA, Kim IY, Kim MA, Lee YS, Park JH, Lee BY, Kim IA, Kim HS, Kim SA, Yoon AR, Yun CO, Kim EY, Lee K, Choi KS (2014). Stronger proteasomal inhibition and higher CHOP induction are responsible for more effective induction of paraptosis by dimethoxycurcumin than curcumin. CELL DEATH DIS.

[45] Zhang P, Gao K, Tang Y, Jin X, An J, Yu H, Wang H, Zhang Y, Wang D, Huang H, Yu L, Wang C (2014). Destruction of DDIT3/CHOP protein by wild-type SPOP but not prostate cancer-associated mutants. HUM MUTAT.

[46] Updegraff BL, O'Donnell KA (2013). Stressing the importance of CHOP in liver cancer. PLOS GENET.

[47] Petiwala SM, Berhe S, Li G, Puthenveetil AG, Rahman O, Nonn L, Johnson JJ (2014). Rosemary (Rosmarinus officinalis) extract modulates CHOP/GADD153 to promote androgen receptor degradation and decreases xenograft tumor growth. PLOS ONE.

