# Supplementary material for: Long noncoding RNA HOXA-AS2 promotes gastric cancer proliferation by epigenetically silencing P21/PLK3/DDIT3 expression
Source: Oncotarget. 2015 Sep 10;6(32):33587–601. doi: 10.18632/oncotarget.5599 (PMC4741787; doi:10.18632/oncotarget.5599)
Supplement: Supplementary file 1 [file oncotarget-06-33587-s001.pdf]

Long noncoding RNA HOXA-AS2 promotes gastric cancer proliferation by epigenetically silencing P21/PLK3/DDIT3 expression

Supplementary Material

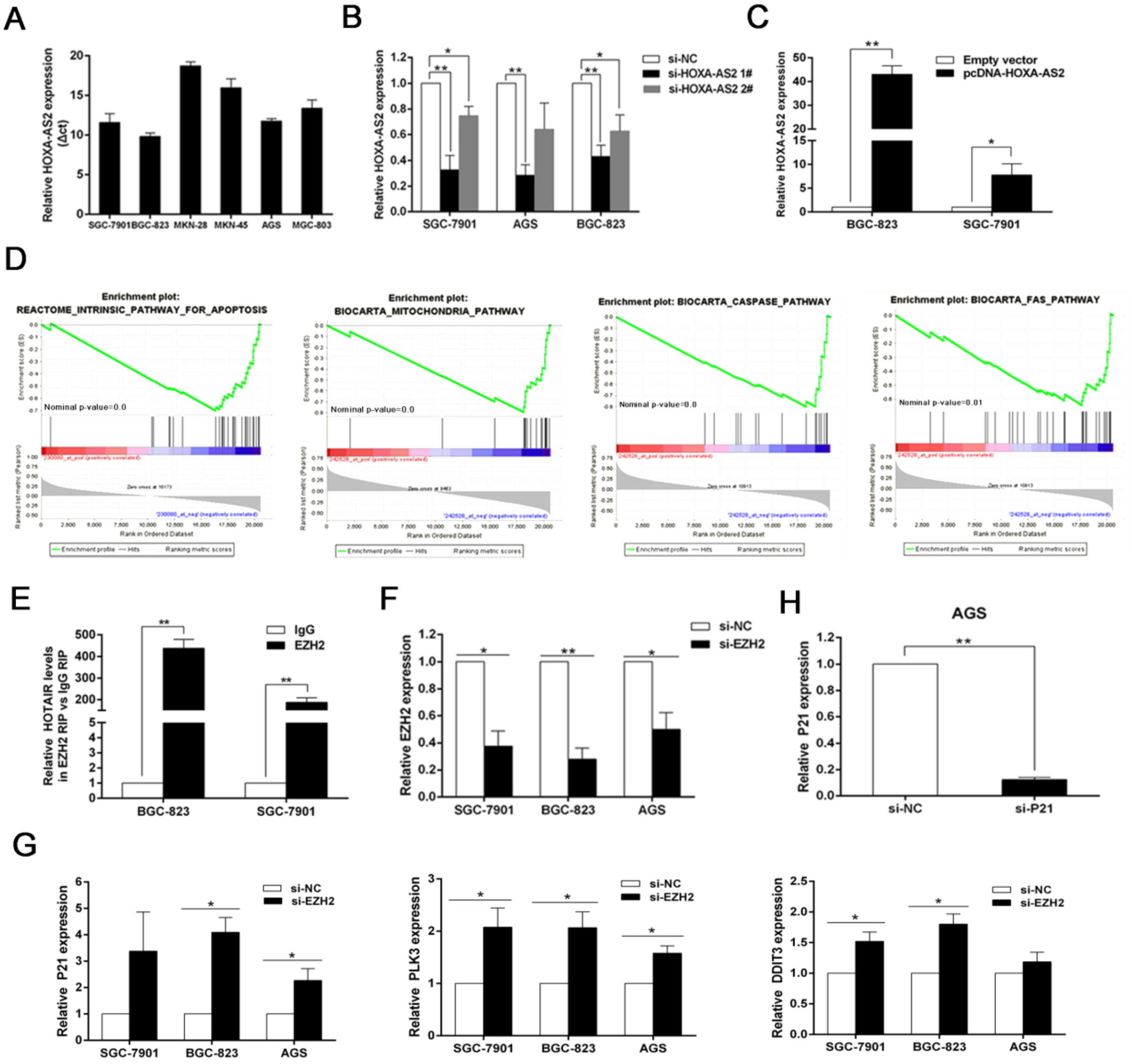

Figure S1. (A) HOXA-AS2 expression levels of GC cell lines (SGC-7901, BGC-823, MKN-28, MKN-45, AGS and MGC-803). Results are shown as ∆ct. (B) Relative expression level of HOXA-AS2 in SGC-7901, AGS and BGC-823 cells transfected with si-NC or si-HOXA-AS2, was tested by qRT-PCR. (C) Relative expression level of HOXA-AS2 in SGC-7901 and BGC-823 cells transfected with empty vector or pcDNA-HOXA-AS2, was tested by qRT-PCR. (D) GSE15459 and GSE51105 analysis indicated that HOXA-AS2 was negatively correlated with intrinsic pathway for apoptosis, mitochondria pathway, caspase pathway and fas pathway. (E) lncRNA HOTAIR was used as positive

control to bind with EZH2 in BGC-823 and SGC-7901 cells. (F) Relative expression level of EZH2 in SGC-7901, BGC-823 and AGS cells, transfected with si-NC or si-EZH2, was tested by qRT-PCR. (G) The levels of P21/PLK3/DDIT3 were determined by qRT-PCR after si-EZH2 was transfected into BGC-823, SGC-7901 and AGS cells. (H) P21 expression level in AGS cells, transfected with si-NC or si-P21, was tested by qRT-PCR. Error bars indicate mean ± standard errors of the mean. *P<0.05, **P<0.01.

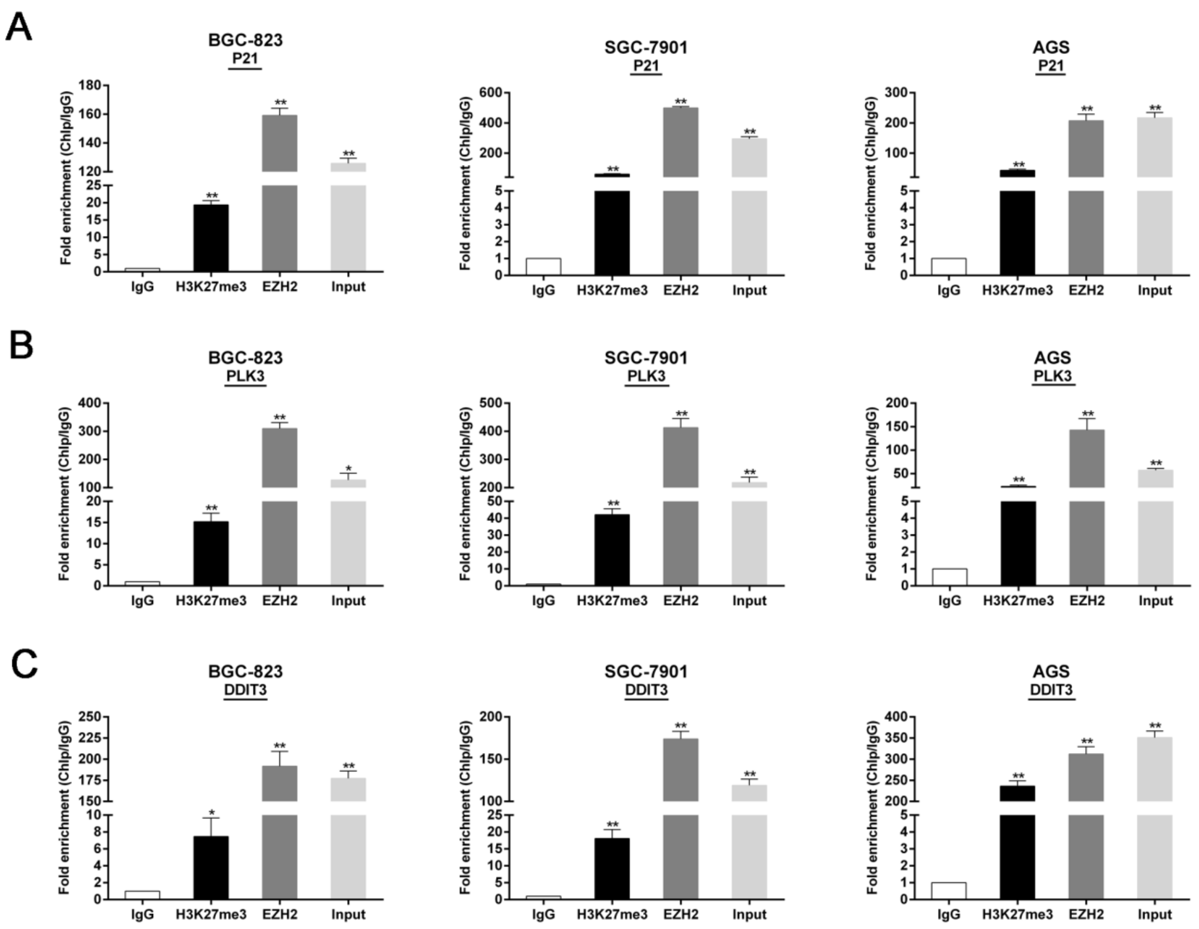

Figure S2. (A, B and C) ChIp-qRT-PCR of EZH2 occupancy and H3K27me3 binding in the P21/PLK3/DDIT3 promoters in BGC-823, SGC-7901, AGS cells; IgG as a negative control. Error bars indicate mean ± standard errors of the mean. *P<0.05, **P<0.01.
